# Guillois Cement for Temporary Fillings

**Published:** 1870-12

**Authors:** 


*Guillois' Cement for Temporary Fillings.*—This cement has lately been introduced to the profession in this country, after a short trial in England where it has been favorably noticed by some respectable practitioners. Chas. J. Fox, M. R. C. S., L. D. S. communicates the following to the "*British Journal of Dental Science* concerning his experience in the use of this material:

"I have been for some time expecting to see some communication respecting this cement, recently introduced, as every one who tries it expresses privately extreme satisfaction with it. When this is the case, I think it is only fair to say so publicly. It is of the same nature as that commonly called osteoplastic, but it differs from it in this particular, that it can be mixed to a consistence much resembling putty, and in that state can be manipulated for some minutes without setting irretrievably. If you mix the other osteoplastics as thick as this, they set rapidly or crumble; if you use them in a thinner condition, they run about on the gums and teeth. When once set it is so hard, if it has been properly manipulated, as to turn the edge of the instrument, should it be deemed requisite to remove it. As to its durability, it is of course impossible to say much, seeing that it has only been introduced into England for a few months; but this much may be said, that taking four months' experience with other cements, and four months' with this, I have found it so superior that I have entirely discarded all other osteoplastics, amalgams, etc. In small cavities in the incisors, or in shallow cavities where osteoplastics would wash out in a short time and dissolve away, Guillois' Cement remains at the end of four months as good as when it was put in. I can not tell what *further* experience may prove, but *so far*—and only for four months' experience do I speak—I have not had one failure, which is more than I can say of any other."

Four shades, two of blue and two of yellow are prepared and indicated by a sample attached to each package.

We advise a trial of it for temporary fillings and should be pleased to hear of the results.

